# Matched-pair analysis of patients with female and male breast cancer: a comparative analysis

**DOI:** 10.1186/1471-2407-11-335

**Published:** 2011-08-04

**Authors:** Robert Foerster, Frank G Foerster, Volkhard Wulff, Birgit Schubotz, Dieter Baaske, Matthias Wolfgarten, Walther C Kuhn, Christian Rudlowski

**Affiliations:** 1 Department of Gynecology and Obstetrics, Center of Integrated Oncology, University Hospital Bonn, Siegmund-Freud-Str.25, 53113 Bonn, Germany; 2 Department of Economical Sciences, University of Applied Sciences, Zwickau, and Outpatient Department of Gynecological Oncology and Palliative Care, Poliklinik GmbH, Chemnitz, Germany; 3 Cancer Register of Southwest Saxony, Zwickau, Germany; 4 Cancer Register of Chemnitz, Chemnitz, Germany; 5 Department of Radiation Oncology, Klinikum Chemnitz, Chemnitz, Germany

## Abstract

**Background:**

Male breast cancer (MBC) is a rare disease accounting for approximately 1% of all breast carcinomas. Presently treatment recommendations are derived from the standards for female breast cancer. However, those approaches might be inadequate because of distinct gender specific differences in tumor biology of breast cancer. This study was planned in order to contrast potential differences between female and male breast cancer in both tumor biological behavior and clinical management.

**Methods:**

MBC diagnosed between 1995-2007 (region Chemnitz/Zwickau, Saxony, Germany) was retrospectively analyzed. Tumor characteristics, treatment and follow-up of the patients were documented. In order to highlight potential differences each MBC was matched with a female counterpart (FBC) that showed accordance in at least eight tumor characteristics (year of diagnosis, age, tumor stage, nodal status, grade, estrogen- and progesterone receptors, HER2 status).

**Results:**

108 male/female matched-pairs were available for survival analyses. In our study men and women with breast cancer had similar disease-free (DFS) and overall (OS) survival. The 5-years DFS was 53.4% (95% CI, range 54.1-66.3) in men respectively 62.6% (95% CI, 63.5-75.3) in women (p > 0.05). The 5-years OS was 71.4% (95% CI, 62.1-72.7%) and 70.3% (95% CI, 32.6-49.6) in women (p > 0.05). In males DFS analyses revealed progesterone receptor expression as the only prognostic relevant factor (p = 0.006). In multivariate analyses for OS both advanced tumor size (p = 0.01) and a lack of progesterone receptor expression were correlated (p = 0.01) with poor patients outcome in MBC.

**Conclusion:**

Our comparative study revealed no survival differences between male and female breast cancer patients and gives evidence that gender is no predictor for survival in breast cancer. This was shown despite of significant gender specific differences in terms of frequency and intensity of systemic therapy in favor to female breast cancer.

## Background

In contrast to the breast cancer in women, male breast carcinoma (MBC) is rare, accounting for less than 1% of all cases of breast carcinoma with an incidence of 1 in 100.000 men [1,2]. In the United States up to 1.500 primary male breast cancer patients per year were identified [3]. Data from the SEER Program of the National Cancer Institute (NCI) showed a slight increase over time from 1.0 per 100,000 in the late 1970s to about 1.2 per 100,000 in 2000-2004 [4]. Age-standardized rates for male breast cancer revealed that the incidence peaked in the year 2000 at 1.24 per 100.000 for men whereas the peak in women was observed in 1999 at 165 per 100.000 women [1]. Because of its rarity, most information about this disease has been obtained from small, single-institutional or retrospective studies or by extrapolation from breast cancer trials in women [5]. Its phenotypic alterations are not well studied, and therapy is mainly based on experiences with female breast cancer [6].

Although both diseases share similarities, there are notable differences reported [7]. Breast cancer in men occurs with higher stage, possibly because of delayed breast cancer detection [2,8]. Male breast cancers also are typically more low grade and frequently hormone receptor--positive [7]. The relatively unfavorable outcome in male breast cancer has been attributed to more advanced local tumor stage and high incidence of lymph node invasion at the time of diagnosis [9-11]. It has been postulated that close proximity to skin and nipple facilitates early invasion of lymph vessels leading to earlier regional and distant metastasis.

Gender-comparative survival studies revealed divergent data on patients outcome [11-16]. This might depend on the lacking adjustment for prognostic relevant parameters in comparative analyses between male and female cohorts. However, specific informations about disease outcome in men has emerged great attention since tailored therapy is of incremental importance in breast cancer.

Several groups have recently reported comparative analyses for male versus female breast cancers, but these studies failed to be adjusted for a variety of prognostic relevant parameters in breast cancer like age, tumor size, nodal stage, tumor grade hormone receptors and HER2 expression [10-16].

To our knowledge this is the first comprehensive gender-specific survival analysis considering all established tumor and patients characteristics established in female breast cancer in order to elucidate potential differences in both disease-free and overall survival.

## Methods

Consecutively in the years 1995 to 2007 113 men were diagnosed with breast cancer in the administrative district of Chemnitz in the State of Saxony, Germany. All data of these patients was obtained from the district's two cancer registers located in the cities of Chemnitz and Zwickau which together oversee a total of about 1.5 million inhabitants. Primary surgery was performed exclusively in community or district hospitals. Adjuvant treatment and follow-up care were carried out additionally in outpatient departments. With the approval of the institutional review boards data regarding patients' age, histology, TNM stage, tumor grade, date of diagnosis, date of metastasizing and date of death were recorded. In addition, detailed information was gathered on estrogen, progesterone and HER2 receptor expression, type of primary surgery, as well as on chemotherapy, endocrine treatment and radiation therapy in the adjuvant and palliative setting. The study was approved by the Ethic Committee of the University of Bonn, Germany. Patient data were collected retrospectively and blinded (name and date of birth). Therefore and in accordance with the Ethic Committee patient consent was not required for the inclusion into the study. For 108 male breast cancer patients one matching woman could be chosen from a total of 13.333 female breast cancer patients. The matching process was based on eight features relevant for breast cancer prognosis i.e. year of diagnosis (within ± 5 year) age, tumor stage, nodal stage, tumor grade, estrogen and progesterone receptor expression, as well as HER2 expression. Five men had to be excluded from the current study because no women with breast cancer could be matched. If more than one female patient was eligible the best match was chosen by random selection. The matching procedure was conducted blinded without any information about patients outcome.

To ensure the comparability of the male with the female patients the chi-square test was applied for every matching criterion. Overall survival (OS) and disease-free survival estimations were calculated according to Kaplan and Meier. Disease-free survival (DFS) was defined as the time period from diagnosis to death of recurrence or death whichever occurred first. Overall survival was considered as the time period from first diagnosis until death. The effect of gender on survival was estimated by hazard ratio (HR) and its 95% confidence interval (95% CI). P values < 0.05 were considered statistically significant. All statistical analyses were performed using SPSS version 17.0.

## Results

### Matching criteria

Based on the above mentioned matching procedure and under consideration of eight matching criteria 108 male/female pairs were available for survival analyses. Table 1 displays the distribution of male and female patients according to the matched tumor characteristics (Table 1). The number of cases in the different clinicopathological subgroups revealed no significant gender specific differences. Median age at diagnosis was 67 years in male and female patients (range, 43--89 years for men and 36-89 years for women). Less than 40.0% of the male patients had early tumor stages (pTis, pT1) whereas 25.0% of the males showed pT4 stages. Four male patients (3.7%) had a ductal carinoma in situ, seven (6.5%) were grade 1. Male patients with lymph node metastasis were found in 43.6%. 10 patients (9.3%) of both gender had primary advanced disease with distant metastasis. Estrogen and progesterone receptor expression was positive in 65.7% and 63.9% respectively. Seven (6.5%) HER2 (3+ immunoscore and/or FISH amplification) positive male tumors could be assigned. No age-related distribution of tumor characteristics could be observed in men. Breast cancer in younger patients (< 50 years) were not associated with tumor parameters representing a more aggressive phenotype like advanced tumor stage, poor differentiation and a lack of hormone receptor expression.

**Table 1:** Matching criteria of male and female breast cancer patients

	Men		Women		Χ^2^
**Median age (range)**	67 (43-89)		67 (36-89)		

< 49	9	8.3%	8	7.4%	**0.98**
50-59	12	11.1%	13	12.0%	
60-69	42	38.9%	40	37.0%	
70-79	30	27.8%	31	28.7%	
> 80	15	13.9%	16	14.8%	
**Tumor Stage **pTis	4	3, 7%	4	3.8%	**0.91**

pT1	37	35.6%	36	34.6%	
pT2	32	30.8%	37	35.6%	
pT3	5	4.8%	4	3.8%	
pT4	26	25.0%	23	22.1%	
**Nodal Stage **pN0	57	56.4%	56	56.0%	**0.85**

pN+	44	43.6%	44	44.0%	
**Grading **G1	7	6.5%	6	6.1%	**0.93**

G2	62	63.3%	62	62.6%	
G3	29	29.6%	31	31.3%	
**ER-expression **ER-	19	17.6%	19	17.6%	**1.0**

ER+	71	65.7%	72	66.7%	
unknown	18	16.7%	17	15.7%	
**PR-expression **PR-	21	19.4%	22	20.4%	**0.86**

PR+	69	63.9%	69	63.9%	
unknown	18	16.7%	17	15.7%	
**HR-expression **HR+	78	72.2%	77	71.3%	**0.84**

HR-	13	12.1%	14	13.0%	
unknown	17	15.7%	17	14.7%	
**HER2 **HER 2 -	70	64.8%	63	58.3%	**0.36**

HER 2 +	7	6.5%	10	9.3%	
unknown	31	28.7%	35	32.4%	

Chi-Quadrat test (X^2^) was calculated in order to demonstrate accordance between male and female matching parameters.

The following patients characteristics could be additionally obtained from patient charts (Table 2): histological type, kind of surgery and adjuvant treatment. Nearly 80% of the tumors in men showed ductal histology whereas almost 60% of female cases had a ductal subtype. It is obvious that male breast cancer was predominantly treated by mastectomy (88.7%) whereas in females the rate of mastectomy was 45.4% (p < 0.001). Axillary dissection was performed in 87.0% of the males and 85.1% of the female patients (p > 0.05).

**Table 2:** Not-matched tumor characteristics and treatment features of female and male breast cancer patients

	Men		Women		χ^2^
**Histology**					
DCIS	4	3.7%	3	2.8%	0.14
Invasive ductal carcinoma	86	79.6%	64	59.8%	
Invasive lobular carcinoma	5	4.6%	16	15.0%	
Others*	13	12.0%	25	23.4%	

**Surgery**					
Mastectomy	94	88.7%	49	45.4%	**0.001**
BCS	12	11.3%	51	47.2%	
No surgery	0	0%	8	7.4%	
Axillary dissection	94	87.0%	92	85.1%	0.76
No axillary dissection	14	13.0%	16	14.8%	

**Adjuvant radiotherapy**					
Radio therapy received	62	60.8%	72	87.8%	**0.001**
No therapy	40	39.2%	10	12, 20%	

**Adjuvant systemic therapy**					
Chemotherapy	15	15%	21	23.6%	**0.005**
Chemo-/Hormone therapy	17	17%	27	30.3%	
Hormone therapy	31	31%	31	34.8%	
Trastuzumab	3	3%	1	1.1%	
No therapy	34	34%	9	10.1%	

*Others: mucinous, scirrhous, squamous, mixed.

More than 60% of the males received adjuvant radiotherapy whereas in almost 90% of the female patients a radiation was documented (p < 0.001). Significant differences regarding adjuvant systemic treatment between male and female patients were observed: 34% of the male patients received no adjuvant systemic treatment whereas only 10.1% of the female patients were without adjuvant therapy (p < 0.005). Adjuvant endocrine therapy was administered in 48.0% (65.1% in females) of the male patients consisting of tamoxifen in 43.2% (36.0% in females), aromatase inhibitors in 4.3% (10.7% in females) and a switch of tamoxifen and aromatase inhibitors in 0.5% (13.8% in females). 4.6% of the female patients receive adjuvant GnRH analogues.

A variety of different adjuvant regimens were documented in male patients. Overall 32.0% of the male patients (53.9% in females) were treated with adjuvant chemotherapy. 11.6% of the patients received CMF (9.3% in females), 11.4% anthracycline containing regimens (34.4% of the females) and 9.0% taxanes (10.2% in females). In 3.0% of the male patients (1.1% in females) a trastuzumab containing therapy was administered.

### Survival analyses

**Figure 1:**
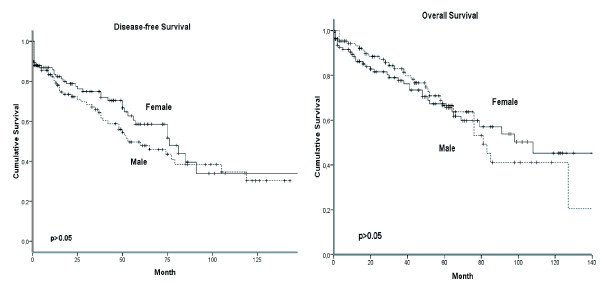
**Gender-specific DFS (a) and OS (b) of the matched-pair study group**.

**Figure 2:**
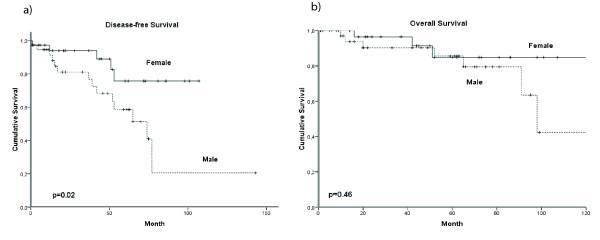
**Gender-specific DFS (a) and OS (b) for tumor stage pT1**. Males with pT1 tumor showed a significantly reduced DFS (p = 0.02).

With a median follow-up time of 56 months (range,1-143 months) for men and 48 months (range, 1-108 months) for women in our study men and women with breast cancer showed similar disease-free (DFS) and overall (OS) survival (Figure 1). 35 (31.0%) male patients suffered from tumor relapse compared with 28 (25.9%) cases in women. The 5-years DFS was 53.4% (95% CI, 54.1-66.3) and 62.6% (95% CI, 63.5-75.3) in men and women, respectively. 36 deaths in men (31.9%) and 32 (29.6%) among women occurred. In both groups 20 (18.5%) of them were attributed to primary cancer. The 5-years OS were 71.41% (95% CI, 62.1-72.7%) and 70.3% (95% CI, 32.6-49.6) in men and women, respectively. None of the 4 patients in each gender group with a carcinoma in situ has relapsed. In general, we found no difference in DFS and OS in the matched pair comparison between male and female breast cancer patients (Table 3). DFS for tumor stage pT1, however, was significantly (p = 0.01) reduced in males as compared to females (Figure 2). It is remarkable that 12/38 (31.6%) male patients in stage pT1 suffered tumor relapse and died disease-specifically.

**Table 3:** Gender-specific DFS and OS according to the matching criteria of the study group

	DFS			OS		
	**Men [median months]**	**Women [median months]**	p-Value*	**Men [median months]**	**Women [median months]**	p-Value*

**Study group**						

**Age **≤ 49	94	91	n.s.	115	127	n.s.
50-59	61	65	n.s.	105	73	n.s.
60-69	79	85	n.s.	108	85	n.s.
70-79	49	51	n.s.	61	65	n.s.
≥ 80	68	39	n.s.	80	51	n.s.
						

**Tumor stage **pT1	69	90	**0.01**	98	127	n.s.
pT2	79	85	n.s.	95	99	n.s.
pT3	50	67	n.s.	76	82	n.s.
pT4	47	32	n.s.	41	39	n.s.
						

**Lymphnodes **pN0	79	85	n.s.	103	106	n.s.
pN+	38	42	n.s.	80	63	n.s.
						

**Metastases **cM0	-	-		99	96	n.s.
cM1	-	-		10	8	n.s.
						

**Grading **G1**	-	-		-	-	
G2	94	88	n.s.	103	90	n.s.
G3	72	76	n.s.	80	78	n.s.
						

**Estrogen receptor **ER-	82	70	n.s.	41	76	n.s.
ER+	81	119	n.s.	108	127	n.s.
Unknown	97	67	n.s.	86	57	
						

**Progesterone receptor **PgR-	38	91	n.s.	38	58	n.s.
PgR+	77	154	n.s.	108	127	n.s.
Unknown	97	75	n.s.	86	76	n.s.
						

**HER2 receptor **HER2-	77	91	n.s.	108	127	n.s.
HER2+	36	57	n.s.	41	68	n.s.

* p-value < 0.05 was calculated to be statistically significant

** none of the patients had tumor relapse or died.

**Figure 3:**
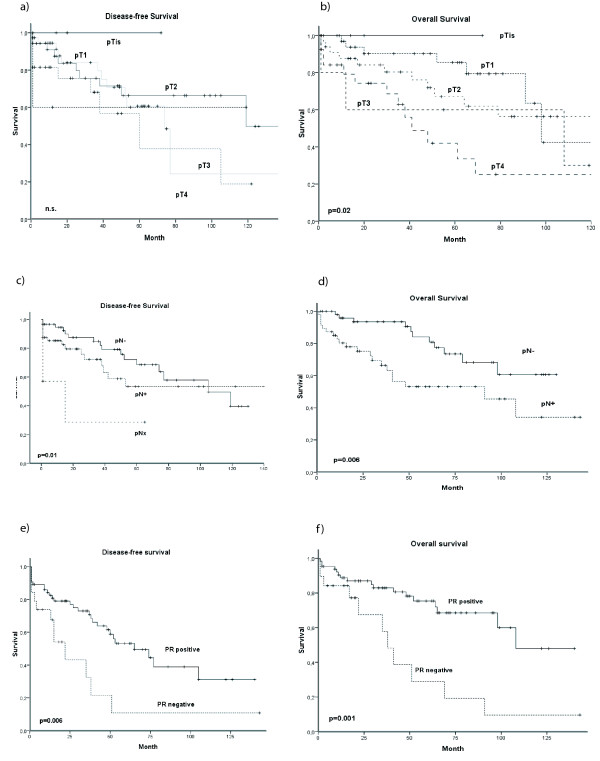
**DFS and OS of male patients according to prognostic relevant tumor characteristics: Tumor (T) stage (a, b), nodal (N) status (c, d), and progesterone receptor (PR) status (e, f)**. p < 0.05 is considered as statistically significant.

In order to elucidate male gender-specific patients outcome in men survival analyses were performed additionally for male patients only (Figure 3). Progesterone receptor expression was the only prognostic relevant factor (p = 0.006, univariate analysis only) for DFS. In addition, univariate analyses revealed tumor stadium, nodal stage and progesterone receptor expressions as statistically significantly associated with OS in male breast cancer. In multivariate analyses for OS both advanced tumor size (p = 0.01) and a lack of progesterone receptor expression (p = 0.03) were correlated with poor patients outcome.

Male breast cancer patient bear a noticeable risk of secondary cancers. Allover, 21 (19.4%) male breast cancer patients had an additional malignant disease, 7 (6.5%) before and 14 (12.9%) after diagnosis of breast cancer. The most frequent second primary cancers were prostate, gastric, colorectal carcinoma and skin cancer.

## Discussion

In the past decade it has emerged great attention to obtain evidences about the clinical outcome of male patients with breast cancer [17-19]. This is due to its rising incidence and the persistent lack of established treatment guidelines. Actually, treatment strategies are derived from female breast cancer [7]. Without evidence-based data to support female-to-male extrapolation, epidemiologic comparisons become an alternative source of information.

A couple of features in male breast cancer are of particular interest. Male breast cancer was more like late-onset than female breast cancer with more than 90% of male patients aged 50 years and older. It is well known that breast cancer in men is diagnosed 5 to 10 years later than in women [20] which also might account for the less aggressive adjuvant treatment modalities given to male patients in this study.

Furthermore, more than 90% of male breast carcinomas were hormone receptor positive whereas less than 10% overexpressed HER2. This is in line with previous findings [21,22] and supports the hypothesis that the luminal like molecular subtype is predominant in men [20,23,24]. As opposed to female breast cancer the progesterone receptor expression in men was an independent prognostic factor in this study.

Male patients had a significant proportion of advanced tumor stages (pT2-4 > 60%) and more than 40% showed nodal involvement [25]. This is at least in part caused by a delay of diagnosis in men of more than six months in average [26-28]. There is little public awareness of breast carcinoma in men and public education regarding the existence of male breast carcinoma. No recommendations for self-examination or examination of the male breast by physicians exist. No guidelines recommend screening mammography at any age for men because of the rarity of the disease.

In male breast cancer adjuvant treatment was administered less frequently. Almost one third of men received no adjuvant treatment as compared to 10% in the matched-pair female group. Only 32% of male patients had adjuvant chemotherapy (54% in women) and less than 50% adjuvant endocrine treatment (65% in women). Nevertheless, our comparative study revealed no survival differences between male and female breast cancer patients and gives evidence that gender is no predictor for neither disease-free nor overall survival. These findings are remarkable considering the different systemic treatment given to male patients as compared to the matched female patients. Due to exact matching procedures with respect to all established clinical and pathological prognostic factors (Table 1), a gender specific bias of prognostic features seems unlikely.

In addition, considering clinically relevant subgroups no significant overall survival differences could be observed between men and women. In our study men with pT1 tumors had significantly worse DFS compared to females. Those male patients with pT1 tumors who have relapsed so far showed no poor prognostic features e.g. all of them were hormone receptor positive. Furthermore, all of them received adjuvant tamoxifen. Therefore, no specific predictors for metastasis in this low risk group could be identified. Potential explanations for a comparatively worse outcome of males with early breast cancer are obviously. There is no evidence that tamoxifen is highly effective in primary male breast cancer patient. Data supporting response to treatment are mainly retrospective or from small trials [29]. There are major concerns regarding patients compliance and tamoxifen metabolic activity which might have negative impact on the efficacy. Clinical response to tamoxifen depends on the biotransformation via the cytochrome P450 isoenzyme CYP2D6 isoform, to the active metabolite endoxifen. CYP2D6 activity can be reduced in men both by genetic variation or concurrent use of drug inhibitors, which can significantly reduce endoxifen plasma concentrations [30]. However, tamoxifen remains the standard therapy in endocrine responsive male breast cancer and clincial and translational studies on this field are needed.

Our cohort study has several limitations and selection bias regarding the match pair analysis could not be excluded. Beside its retrospective character the number of male patients is small compared to female studies. Therefore analyses especially of patients subgroups has limited clinical significancy.

Several other groups compared male and female breast cancer features and prognosis [10,11,15,31-33] with divergent results. Marchal and colleagues found similar DFS but worse OS for male as compared to female breast cancer patients in a retrospective study [11]. The authors explained the OS difference with a higher amount of comorbidities in men. However, the study was based on matched-pair analyses of only 58 male breast carcinomas and three matching criteria (age, year of diagnosis, stage). Tumor differentiation, hormone receptor status and HER2 positivity were not considered. This might reflect an incomplete matching procedure and could better explain the divergent findings to our results.

## Conclusion

Our retrospective study showed no disease and overall survival differences between male and female breast cancer patients matched for year of diagnosis, age, tumor size, nodal stage, tumor grade, estrogen receptor, progesterone receptor and HER2 expression. This is of great importance since male patients receive obviously less aggressive adjuvant treatment.comparted their female matched pairs. A significantly reduced DFS in male with pT1 tumors was found. In multivariate analyses only early stage and progesterone receptor positvity were statistically significant related to improved overall survival in male breast cancer.

## Abbreviations

CI: Confidence Interval; CMF: Cyclophosphamide/Methotrexate/Flourouracil; DFS: Disease-free Survival; FBC: Female Breast Cancer; FISH: Fluorescence in-situ Hybridisation; GnRH: Gonadotropin Releasing Hormone; MBC: Male Breast Cancer; OS: Overall Survival.

## Competing interests

The authors declare that they have no competing interests.

## Authors' contributions

RF, FGF and CR conceived, planned and designed the study. VW and DB performed the statistical analysis. MW, BS and WCK participated participated in its design and coordination and helped to draft the manuscript. All authors read and approved the final manuscript.

## Pre-publication history

The pre-publication history for this paper can be accessed here:

http://www.biomedcentral.com/1471-2407/11/335/prepub
